# Considering trauma exposure in the context of genetics studies of posttraumatic stress disorder: a systematic review

**DOI:** 10.1186/2045-5380-3-2

**Published:** 2013-01-03

**Authors:** Julia DiGangi, Guia Guffanti, Katie A McLaughlin, Karestan C Koenen

**Affiliations:** 1 DePaul University, Chicago, IL, USA; 2 Department of Psychiatry, Columbia University/New York State Psychiatric Institute, New York, NY, USA; 3 Division of General Pediatrics, Department of Psychiatry, Boston Children’s Hospital, Harvard Medical School, Boston, MA, USA; 4 Department of Epidemiology, Mailman School of Public Health, New York, NY, USA

**Keywords:** Posttraumatic stress disorder, Trauma, Genetics, Genome-wide association studies, Gene–environment interaction

## Abstract

**Background:**

Posttraumatic stress disorder (PTSD) is a debilitating anxiety disorder. Surveys of the general population suggest that while 50-85% of Americans will experience a traumatic event in their lifetime, only 2-50% will develop PTSD. Why some individuals develop PTSD following trauma exposure while others remain resilient is a central question in the field of trauma research. For more than half a century, the role of genetic influences on PTSD has been considered as a potential vulnerability factor. However, despite the exponential growth of molecular genetic studies over the past decade, limited progress has been made in identifying true genetic variants for PTSD.

**Methods:**

In an attempt to aid future genome wide association studies (GWAS), this paper presents a systematic review of 28 genetic association studies of PTSD. Inclusion criteria required that 1) all participants were exposed to Criterion A traumatic events, 2) polymorphisms of relevant genes were genotyped and assessed in relation to participants’ PTSD status, 3) quantitative methods were used, and 4) articles were published in English and in peer-reviewed journals. In the examination of these 28 studies, particular attention was given to variables related to trauma exposure (e.g. number of traumas, type of trauma).

**Results:**

Results indicated that most articles did not report on the GxE interaction in the context of PTSD or present data on the main effects of E despite having data available. Furthermore, some studies that did consider the GxE interaction had significant findings, underscoring the importance of examining how genotypes can modify the effect of trauma on PTSD. Additionally, results indicated that only a small number of genes continue to be studied and that there were marked differences in methodologies across studies, which subsequently limited robust conclusions.

**Conclusions:**

As trauma exposure is a necessary condition for the PTSD diagnosis, this paper identifies gaps in the current literature as well as provides recommendations for how future GWAS studies can most effectively incorporate trauma exposure data in both the design and analysis phases of studies.

## Review

**Figure 1:**
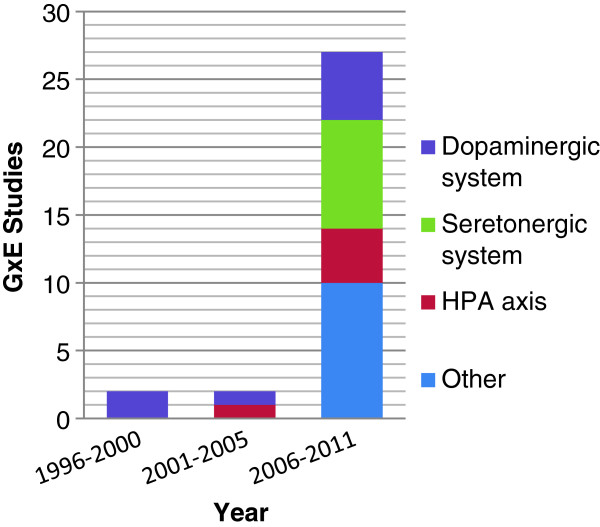
GxE PTSD studies published by year and neurobiological system.

Post-traumatic stress disorder (PTSD) occurs following exposure to a traumatic event and is defined by distinct symptom clusters of re-experiencing, avoidance and numbing, and arousal persisting for more than 1 month after trauma [1]. At least 1 in 9 American women and 1 in 20 American men will meet criteria for the diagnosis in their lifetime [2,3]. Among the 50% to 85% of Americans who are exposed to a traumatic event, the conditional risk of PTSD ranges from 2% to 50% [3-5]. Why some individuals develop PTSD following trauma exposure while others are resilient remains a key question in trauma research. The importance of genetic influences on PTSD risk have been recognized for half a century [6] and heritability estimates range from approximately 30 – 70% [7-9]. However, the first molecular genetic study of PTSD was not published until 1991 [10]. Limited progress has been made in identifying true or causal genetic variants for PTSD despite the fact that the number of molecular genetic studies of PTSD has grown exponentially in the past decade (Figure 1). Elsewhere, we have reviewed the state and limitations of genetic research on PTSD. We have discussed how these limitations can be addressed through genome-wide association studies (GWAS), which combined with well-powered replication samples, offer the best opportunity to identify novel “true” risk variants for the disorder [11]. Large-scale GWAS of PTSD are now underway.

This paper presents a systematic review of molecular genetic studies of PTSD to identify the characteristics of trauma exposure that may be most important to consider in GWAS studies of PTSD. Because trauma exposure is a necessary condition for a PTSD diagnosis, GWAS studies of PTSD will need to contend with how to make best use of trauma exposure data in both the design and analysis phases of their studies. In doing so, GWAS of PTSD will be delving into uncharted territory. Several approaches for examining GxE in the context of GWAS have been proposed [12-14]. However, GWAS of psychiatric disorders have thus far not included consideration of environmental risk factors. This is true even of GWAS for major depression [15-21], where the contribution of environmental determinants such as stressful life events [22] has been well-established. In fact, the authors of a recent mega-analysis of GWAS in major depression concluded, “It is possible that MDD can only be understood if genetic and environmental risk factors are modeled simultaneously (pg. 7)” [23].

Informed by this observation, we thus focused on the most salient design and analysis considerations for GWAS of PTSD. In particular, we focused on extracting information related to three trauma characteristics most known to be associated with PTSD risk. We assessed how these variables were considered in previous studies and how such consideration influenced the study findings. First is the type of trauma, as the conditional risk of PTSD varies by event. For example, assaultive violence events tend to have the general highest conditional risk of PTSD [24]. Based on gender, combat exposure for men, and rape and sexual molestation for women are the event types most strongly associated with PTSD onset [3]. Second, we focused on the number of traumas. Research has indicated that prior exposure to trauma creates greater risk of PTSD from subsequent trauma [4]. Third, we focused on how long ago the traumatic event took place and where the event(s) occurred along the developmental continuum (i.e., whether it occurred in childhood or adulthood). There is evidence to suggest that abuse experienced during childhood places individuals at greatest risk for the development of psychopathology, including PTSD, as adults [25,26].

## Methods

The systematic review was based on articles found in PubMed, PsycINFO and Published International Literature on Traumatic Stress (PILOTS). Inclusion criteria required that 1) all participants were exposed to Criterion A traumatic events, 2) polymorphisms of relevant genes were genotyped and assessed in relation to participants’ PTSD status, 3) quantitative methods were used, and 4) articles were published in English and in peer-reviewed journals. Search terms were based on descriptor headings, which are selected by each of the databases to best describe the subject of the articles. Terms included: posttraumatic stress disorder, PTSD, genetics, and behavioral genetics. Boolean methods were also used in order to find articles that combined the subjects of PTSD and genetics. Although publication date was not constrained, no articles published prior to 1996 met inclusion criteria.

In addition to sample size and demographic characteristics, the following information was extracted from each article that met our inclusion criteria: the type of trauma and whether it occurred in childhood or adulthood, the time since the trauma, whether multiple traumas were assessed and considered in the analysis, and the major findings.

## Results

A total of 28 articles met inclusion criteria (Table 1).

**Table 1:** Demographic information, results and conclusions for GxE Studies of PTSD

**Reference (year)**	**Sample size (% PTSD Cases)**^**a**^	**Mean age (*SD*)**	**Race**	**Primary trauma type**	**Issue of multiple traumas addressed?**	**Gene**	**Significant main effect of trauma?**	**Significant main effect of gene?**	**GxE interaction?**	**General conclusions**
**Studies of Child Trauma on Child PTSD (2)**										
Amstadter et al. (2011)	103; PTSD-RI M= 24.09 *(12.2)*	14.63 *(3.2*)	40.8% EA; 45.6% AA; 13.6% O	Physical injury	NR^b^	*CRHR1*	Yes	Yes	NR	*rs12944712* was significantly related to higher acute PTSD Sxs and increasing trajectory of Sxs over time.
Drury et al. (2009)	88 (NR)	3-6(*NR*)	56% AA; 40% EA	Hurricane Katrina	NR	*DAT1*	NR	Yes	NR	The *9* allele increased risk of PTSD—both in the form of total and Criterion D Sxs.
**Studies of Adults with History of Child and/or Adult Trauma on Adult PTSD (4)**										
Binder et al. (2008)`	900^c^	40.8 (*13.8*)	95.2% AA;	Child abuse and non-child abuse	Yes	*FKBP5*	Yes	No	Yes	Significant interaction between *FKBP5* polymorphisms and child abuse found for adult PTSD Sxs. The interaction for adult trauma was not significant.
			2.2% EA;							
			0.6% L;							
			0.1% A;							
			0.9% Mixed;							
			1.0% Other							
Nelson et al. (2009)	259 (17.8% LT)	NR	NR	Child abuse	NR	*GABRA2*	NR	No	Yes	Interactions b/w child trauma and SNP genotype provide consistent support for GxE interactions involving child trauma and SNP genotype. When separate variables were coded for the presence of one or two risk-associated alleles, significant Gx E interactions are only found for homozygous individuals.
Xie et al. (2010)	2427 (14.0% LT)	38.6 (*10.8*)	47.1% EA; 52.9% AA	Child adversity	Yes	*FKBP5*	Yes	No	Yes	In AAs, the interaction between child adversity and all 4 *FKBP5* SNPs were associated with PTSD. SNP *rs9470080* had strongest conditional effect; for AAs without child adversity, those homozygous for *T* allele had lowest risk of PTSD, while homogygotes with adversity had highest risk.
Xie et al. (2009)	1252 (18.3% LT)	38.9( *11*)	46.5% EA; 53.5% AA	Both	Yes	*SLC6A4*	Yes	No	Yes	*5-HTTLPR* polymorphism alone did not predict PTSD; however it interacted with adult traumatic events and child adversity to increase the risk for PTSD, especially for those with high rates of both types of trauma exposure.
**Studies of Adults which Assessed Adult Trauma (22)**										
Amstadter et al. (2009)	607 (3.6%)	22.6% ≤ 59	90% EA; 3.9% AA; 3.9% L; 1.7% Other; 0.5% Missing	2004 FL hurricanes	Yes	*RGS2*	No	No	Yes	GxE interaction such that *rs4606* moderated risk of PTSD Sxs under high E stress and low social support.
	Both LT and CT Sxs asses’d									
		77.4% ≥ 60								
Kolassa et al. (2010a)	424 (80.2% LT; 48.8% CT)	34.8 (*5.8*)	100% Hutu or Tutsi	Rwandan genocide	Yes	*COMT*	Yes, LT PTSD	No, for LT and CT PTSD	Yes, LT PTSD	*COMT* genotype affected PTSD such that met/met homozygotes had higher risk for PTSD than those with Val allele independent of severity of traumatic load.
							No, CT PTSD		No, CT PTSD	
Kolassa et al. (2010b)	408 (81.1% LT)	34.68 (*5.9*)	100% Rwandan refugees	Rwandan genocide	Yes	*SLC6A4*	Yes , LT PTSD	Yes , LT PTSD	Yes , LT PTSD	Probability of developing PTSD was 100% for *s* homozygotes and there was no dose–response relationship between trauma and PTSD. However, when trauma approached extreme levels, genotype effect disappeared and PTSD approached 100%.
Kilpatrick et al. (2007)	589 (3.2% CT)	22.6%≤ 59	90% EA; 3.9% AA; 3.9% L; 1.7% Other; 0.5% Missing	2004 FL hurricanes	Yes	*SLC6A4*	No	No	Yes	*5-HTTLPR* increased risk of PTSD under low social support condition.
		76.6% ≥ 60								
Thakur et al. (2009)	41 (59% “acute” PTSD)	32 (*NR)*	95% EA; 5% Other	MVA^d^	Yes	*SLC6A4*	NR	Yes	NR	Higher chronic PTSD was found in *ll* genotypes than *sl* and *ss* genotypes.
Dragan al. (2009)	107 (22.4% CT)	35.57 (*12.89*)	NR	Polish flood	Yes	*DRD4*	Yes	Yes	No	At least 1 copy of *DRD4* long allele related to higher total PTSD and Avoidance/Numbing Sxs.
Comings et al. (1996)	56 (66%*)	43.6 (NR)	100% EA	Vietnam War	No	*DRD2*	NR	Yes	NR	59.5% of those with PTSD had *D*_*2*_*A1* allele; of the group that did not have PTSD, only 5.3% had *D*_*2*_*A1* allele.
Bachmann et al. (2005)	160 (73.8%*)	55.7 (*4.2*)	NR	Vietnam War	No	*GCCR*	NR	No	NR	*N363S* and *Bcl*l GR polymorphisms not more frequent in PTSD patients than controls.
Gelernter et al. (1999)	139 (37.4%*)	With PTSD: 44.6 *(3.6)*	100% EA	Vietnam War	No	*DRD2*	NR	No	NR	No allelic association between *DRD2 TaqI “A”* system alleles and PTSD.
		Without PTSD: NR								
Grabe et al. (2009)	1,663 (4.03% LT)	With ≥ 1 traumatic experience: 57.6 (*15.6*); without traumatic experience: 50.0 (*13.3*)	100% EA	Community based sample; variety of events	Yes	*SLC6A4*	Yes	Yes	Yes	GxE interaction found between high expression of *L*_*A*_ allele and frequent trauma.
Koenen et al. (2009)	590 (3.2% CT)	<60 = 22.7%	90.7% EA; 9.5% Other	2004 FL hurricanes	NR	*SLC6A4*	Yes	No	Yes	County-level crime and employment rate modified association between genotype and PTSD risk. The *s’* allele associated with decreased risk in low-risk environments and increased risk in high-risk environments.
Mellman et al. (2009)	118 (47% LT)	39.9(*16.3*)	NR	Various	NR	*SLC6A4*	NR	Yes	NR	*5HT2A G* allele significantly associated with PTSD.
Mustapic et al. (2007)	167 (85% CT and LT)	With PTSD: 40.3 (*7.2*); Without PTSD 38.12 (*4.2*)	100% Croatian Caucasian	Combat-related trauma	No	*DBH*	NR	Yes	NR	PTSD associated with significantly lower plasma *DBH* activity in those carrying *CC* genotype.
Sayin et al. (2010)	77 (23.3% CT and 50.0% LT)	NR	NR	Mild physical trauma	NR	*SLC6A4*	Yes	No	No	Having *L* allele for *5-HTT* gene- linked polymorphic region may cause milder hyperarousal symptoms in those patients who have developed PTSD.
Segman et al. (2002)	206 (50.5% CT)	With PTSD: 39.7 (*11.7*); Without PTSD: 33.9 (*10.2*)	100% Jewish of definite Ashkenazi or non-Ashkenazi origin	Various (e.g., road accidents, terrorism)	NR	*DAT1*	NR	Yes	NR	The nine repeat allele at the *DAT1* locus associated with increased risk for PTSD.
Bailey et al. (2010)	200 (36.5%*^e^)	NR	100% Armenian	1988 Armenian Earthquake	NR	*DRD2, DAT1*	NR	No	NR	Neither *DRD2* nor *DAT1* associated with PTSD.
Sarapas et al. (2011)	40 (50% total; both CT and LT assessed)	With PTSD: 57.30 (13.2); Without PTSD: 51.20 (15.9)	100% EA	9/11 attacks	Yes	*FKBP5*	Yes, child trauma	No	NR	Comparison of LT versus CT PTSD identified overlapping genes with altered expression suggesting enduring markers, while some markers present only in CT PTSD may reflect state measures. As a follow-up, direct comparisons of expression in CT PTSD, LT-only PTSD, and control groups identified *FKBP5* and MHC Class II as state markers, and also identified several trait markers. An analysis of indirect effects revealed that homozygosity for any of 4 PTSD risk-related polymorphisms at *FKBP5* predicted *FKBP5* expression, which mediated indirect effects of genotype on plasma cortisol and PTSD severity.
							Yes, other trauma			
Valente et al. (2011a)	99 (66.5%*)	With PTSD: 37.9 (8.7); Without PTSD: 44 (13.8)	NR	Urban violence	Yes	*COMT*	No, child trauma	Yes	NR	Found significant association (between *met* allele and PTSD in victims of violence
Valente et al. (2011b)	99 (66.5%*)	With PTSD: 37.9 (8.7); Without PTSD: 44 (13.8)	NR	Being victim of an urban violence that could be characterized as criterion A	Yes	*BDNF, DAT1, SLC6A4*	No	Yes, *DAT1*	NR	Only the nine repeat allele of the *DAT1* was associated with an increased risk of PTSD after being exposed to urban violence.
								No, *SLC6A4*		
Hauer et al. (2011)	126 (11.9% CT)	Homozygotes: 67.1(*10.8*); Heterozygotes: 65.8(*9.3*)	NR	Cardiac surgery	NR	*GCCR*	No	Yes	NR	Homozygous *Bcl*l *G carriers at an increased risk for PTSD stress.
Ressler et al. (2011)	NR	NR	Majority AA	NR	NR	*PACAP, PAC1*	NR	Yes, women only	NR	Alterations in the *PACAP–PAC1* pathway involved in abnormal and sex-specific stress responses underlying PTSD. These sex-specific effects may occur via oestrogen regulation of *ADCYAP1R1*.
Tang et al. (2010)	227 (30.4% CT)	43.9 (*12.8*)	100% AA	NR	NR	*DBH*	ME for adult; No ME for child	No	NR	No relationship between *sDβH* and PTSD (i.e., Sx or Dx)

^a^ In the absence of information on percentage of PTSD in sample, M(*SD*) of PTSD symptoms provided, when available.

^b^ NR = Not reported.

^c^ Overall M(*SD)* not reported.

^d^ MVA = Motor vehicle accident.

^e^* = Authors did not distinguish between lifetime (LT) and current (CT) PTSD.

The sample sizes ranged widely from N=40 to 2,427. In fact, seven studies (25%) had sample sizes less than 100 and an additional 14 (50%) had samples between 100 and 500.

### Age

Out of 28 studies, 20 provided age information for both trauma and control groups. Of the studies reporting age, only two focused on child trauma with child PTSD symptoms, with one study focusing on preschoolers who lived through Hurricane Katrina [27] and the other focusing on adolescents who were hospitalized following physical injury [28]. Four studies examined the effects of either 1) the combination of either childhood or adult trauma on adult PTSD or 2) only childhood trauma on adult PTSD. The remaining 22 articles had exclusively adult samples. The adult samples largely had a mean age ranging from 33.9 to 57.6. The exception were two articles on the 2004 Florida Hurricane, which focused on an older adult population; in both these studies more than 76% were over 60 years of age [29,30]. It is difficult to make meaningful comparisons between the results of child vs. adult studies as there are only two studies that focused exclusively on child trauma and its effect on child PTSD. The outcome variable for the remaining studies was adult PTSD.

### Ethnicity

Of studies that reported racial/ethnic information, 56% of the participants were Caucasians, 42% were African American and 2% were classified as Other (e.g., Latino, Asian).

### Measurement of environment (i.e., trauma)

**Figure 2:**
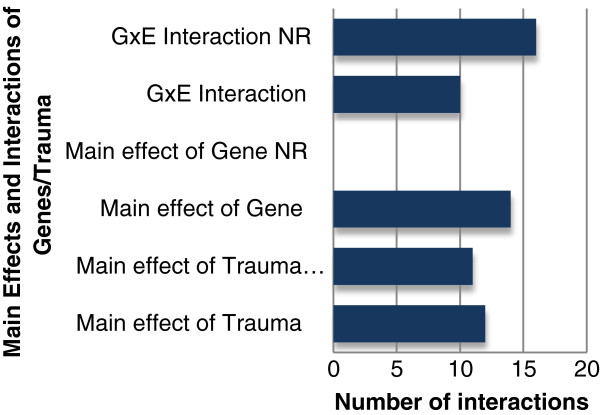
Number of main effects and interactions for genes and environment.

Measurement of trauma varied considerably across studies. There were substantial discrepancies in how trauma was measured in terms of quantity, timing and type. For example, 13 studies had samples that were traumatized multiple times. Of these, eight found a significant GxE interaction, while only one did not; the four remaining studies did not report on the GxE interaction. Furthermore, the majority of articles did not examine the main effect of trauma on PTSD. Out of the 28 articles, 17 articles reported if there was a main effect of trauma on PTSD. Of those, 12 found a significant main effect for trauma (Figure 2).

### Primary trauma type

The level of information provided about the type of primary trauma varied. Four (14%) of the articles selected samples based on exposure to a specific traumatic event such as a natural disaster or physical injury resulting in medical care. For these four studies, all participants had experienced the same traumatic stressor. However, the remaining 24 (86%) articles were studies of community-based samples exposed to a range of traumatic events. Of the articles on adult samples, only 2 (7%) considered events that occurred in both childhood and adulthood as the primary types of trauma. All four studies that examined child abuse/adversity as the primary trauma type found a significant GxE interaction. Likewise, the two studies that examined the Rwandan genocide, and the two studies that studied the Florida hurricanes found a significant GxE interaction.

### Time since the trauma

Information about the amount of time that had elapsed since the traumatic event was provided by 9 (31%) of the articles and, therefore, not included in Table 1. The articles that reported this information typically selected participants based on exposure to a one specific event, such as the 2004 Florida Hurricane Study, Polish flood, Rwandan genocide, or recruited participants following a specific type of event, such as physical injury requiring hospitalization. For the majority of these studies, the index trauma occurred within a year of assessment. The exception to this was the studies of Rwandan genocide survivors; the genocide occurred approximately 12 years prior to assessment.

### Measurement of phenotype

Only 18 articles reported whether participants had current or lifetime PTSD. Of those, seven examined only participants who had current PTSD. Of the studies that reported on lifetime PTSD status and the GxE interaction, all but one found a significant GxE relationship. Of the six studies that assessed for current PTSD, three found a significant GxE interaction.

### Measurement of genotype and neurobiological system

**Figure 3:**
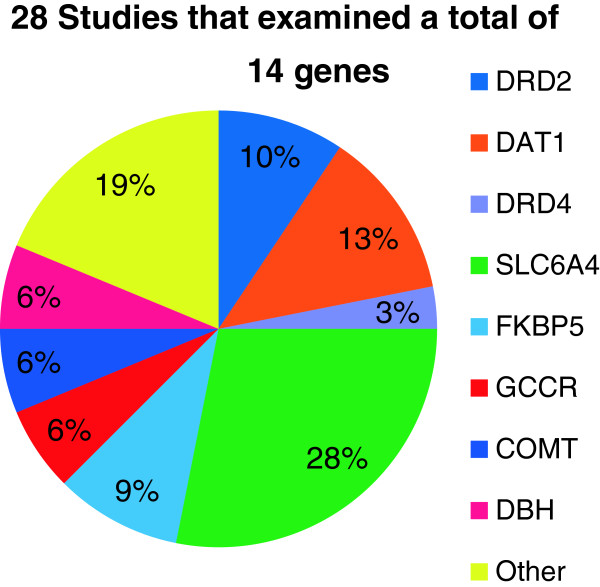
Genes examined by percentage.

All 28 studies assessed for the main effect of gene(s), with 14 studies reporting a significant finding (Figure 2). Within these 28 studies, a total of 14 distinct genes were examined. However, 64.3% of studies focused on the role of four genes: *SLC6A4, DAT1, DRD2,* and *FKBP5*. Examples of other genes examined included *DRD4* and *GCCR* (See Figure 3 for more examples). Of the 14 genes examined, the majority related to the functioning HPA axis, dopaminergic and serotonergic systems. In recent years, genes involved in the HPA axis and the regulation of other neurobiological pathways have received the most attention (Figure 3).

### GxE Findings

Of the studies that examined the statistical relationship between GxE, 10 of the studies reported a significant GxE interaction (Figure 2). Neither of the two studies that had samples entirely comprised of children reported on the statistical GxE relationship, whereas all four of the studies that explored the combined effects of child and adult trauma found a significant interaction. Of the studies that explored the GxE interaction, a significant relationship was found in nine of the studies that examined *SLC6A4*, four for *DAT1*, three for *DRD2* and three for *FKPB5*. Out of the 28 studies, 16 did not report on the statistical GxE relationship. Also, none of the genes that was explored by multiple studies contained information about the GxE relationship in all of the studies in which it was explored. For example, although *FKBP5* was examined in three studies, only two of these three studies explored the GxE relationship [31-33]. Moreover, some of the most explored genes (i.e., *DRD2*) have no information in any of the articles about the statistical interaction between GxE.

## Conclusions

### General conclusions

We systematically reviewed genetic studies of PTSD to identify the most important characteristics of trauma exposure to consider in future GWAS of PTSD. Although we [11,34-36] and others [37-39] have previously reviewed genetic studies of PTSD, this is the first review to explicitly emphasize the GxE relationship. Our review of the 28 available genetic association studies leads to four central conclusions.

First, although the data were often available, most articles did not report on the GxE interaction in the context of PTSD or present data on the main effects of E. Out of 28 studies, only 12 analyzed this interaction, with 10 finding a significant GxE relationship (Figure 2). Although all 28 articles reported the main effect of gene on PTSD, only 17 reported on the main effect of trauma. In the 16 articles that did not examine the GxE interaction, the authors focused solely on the results for the putatively relevant genetic loci. For example, one study examined no interactions and only sought to examine the main effect of gene on PTSD [40]. Similar analytic strategies were used in numerous other studies [41-43]. The lack of systematically presented information on main effect of trauma exposure makes it challenging to reach substantive conclusions about GxE interactions in PTSD based on extant studies.

Second, other studies highlight the importance of considering the GxE interaction when exploring PTSD’s etiology [32]. One study [32] found that in an African American sample, the nature of the interaction between childhood adversity and *FKBP5* SNPs on the development of PTSD depended on environmental conditions. Specifically, for African Americans without child abuse, those homozygous for T allele of *rs9470080* had the lowest chance of developing PTSD. Conversely, however, homozygotes for the same allele who had experienced child abuse had highest risk of developing PTSD. Other studies also found no main genetic effect but found a significant GxE effect on PTSD [29]. Such studies underscore how analysis of the GxE relationship is imperative for gaining a more robust understanding of PTSD’s pathogenesis. Although these findings raise important questions for future research, there is presently insufficient evidence to draw broad conclusions about how genotypes modify the effect of trauma on PTSD.

Third, although molecular genetic studies of PTSD date back to 1991 [10], our review indicated that only a small number of genes have been studied. Across this body of 28 studies, a total of 14 distinct genes have been examined. In 2010, Cornelis and colleagues [11] published a review of genetic research on PTSD. Our review builds on this earlier paper in that it not only includes articles published after 2010, but additionally—and unlike the Cornelis review—only includes studies where both the PTSD and non-PSTD controls were trauma exposed. Since the publication of the Cornelis and colleagues’ review, a total of nine new studies met our criteria and were included. Of all studies reviewed, 64.3% of studies focused on the role of four genes: *SLC6A4, DAT1, DRD2,* and *FKBP5*[33,44-46]. Since the Cornelis review, only three additional genes have been examined. These include: *CRHR1*[28], *GCCR*[47], and *PACAP*[48]. The neurobiological systems that these 14 genes play a role in regulating involve the HPA axis, dopaminergic and serotonergic systems. Interestingly, none of the nine new studies focused on the dopaminergic and serotonergic systems; all focused on the HPA axis and other neurobiological pathways (Figure 1). Although there have been intensive research efforts during the past few decades, the state of the literature remains too preliminary to make substantive conclusions on how genes influence PTSD. As we continue to examine the genetic mechanisms underlying PTSD’s etiology, it is believed that GWAS studies will be an important step forward in this process.

GWAS allows for a comprehensive scan of the genetic risk landscape in an unbiased manner that is untethered to the more traditional and literature-based selection of candidate genes. Thus, GWAS provides a critical hypothesis-generating tool in the identification of genes previously unrecognized in the etiology of PTSD. As the study of genetic risk in PTSD remains in its infancy, the study of genetic variants will be substantially aided by the extended genomic coverage offered by GWAS. GWAS offers great benefit primarily through its use of large numbers of common genetic variants that can aid in the identification of relevant biological mechanisms of the disease. Lately, several approaches have been proposed to facilitate the translation of genetic association results into hypotheses suitable for further investigation. Examples include the identification of polygenic models to study the common contribution of multiple loci to the risk of the disease [49] as well as network-based approaches to leverage models of cell regulation and GWAS data to develop integrative network-based association studies [50]. Finally, to better characterize the functional relevance of genetic association results, the integration of common variants with neurobiological data derived from related experiments on the transcriptome and epigenome of the disease may further our understanding of the pathogenesis of PTSD.

The fourth conclusion of our review relates to core methodological issues that beleaguer this body of literature. Consequently, we are presently unable to draw substantive conclusions about how GxE factors consistently affect PTSD. Distilling information across studies, we are, however, able to describe limitations in this body of work and then offer steps for how these limitations can be addressed in future studies. Specifically, the three most pervasive limitations relate to: 1) measurement of trauma, 2) ascertainment of PTSD cases, and 2) sample size. Although the issue of heterogeneity in trauma research is neither new nor simple, it continues to stymie our understanding of how trauma interfaces with PTSD. Much previous research has demonstrated that the conditional risk of developing PTSD is dependent on the nature of the trauma. For example, a meta-analysis by Ozer and colleagues [51] found that different traumas are associated with different conditional risks. Such conclusions—which demonstrate that all traumas are not created equal—subsequently highlight the inherent problem with treating disparate traumatic events as similar points of comparison. In the current review, not only are there comparisons of diverse traumatic events across studies (e.g., flood [52] vs. genocide [53]), but there is also considerable variability within studies. For example, Valente and colleagues [54] grouped many forms of trauma within a single study without discrimination (e.g., robbery, domestic violence, witnessing violence).

Furthermore, in addition to different events being associated with different conditional risk, research has similarly demonstrated that psychopathology is also dependent on the duration of the traumatic event(s). Chronic trauma exposure, for example, has been associated with greater psychopathology than an acute exposure [55]. Despite this information, only 17 studies assessed for multiple traumas. Moreover, only one study assessed for a dose–response relationship [44].

Just as chronicity of trauma affects symptom constellations, so too does the timing of trauma along the developmental continuum (i.e., childhood vs. adulthood). The vast majority of studies did not examine whether GxE effects differed across the developmental continuum. In fact, only four studies assessed how childhood trauma interacted with genetics to predict adult PTSD symptoms [31,32,56,57]. Examining GxE effects according to developmental timing of trauma exposure is important not only because the association between particular genes and PTSD may vary across development but also because exposure to childhood trauma may heighten risk for onset of PTSD following secondary trauma [25]. For example, with regard to developmental timing, although Binder and colleagues [31] found no main effects of *FKBP5* SNPs on PTSD, they did find a significant interaction between four *FKBP5* SNPs and severity of child abuse on adult PTSD symptoms. Interestingly, while none of these four articles found a main effect for genotype, all found a significant GxE interaction, again underscoring how analysis of the GxE relationship is imperative for gaining a more robust understanding of PTSD’s etiology.

As trauma is a necessary precursor to PTSD, it follows that a lack of continuity in our operationalization of trauma would cause similar disruptions in our understanding of PTSD. Caseness of a PTSD is another serious confound that limits the breadth of our conclusions. Across these 28 studies, individuals selected into the PTSD case group likely had a mix of PTSD statuses. While some individuals suffered from chronic or acute PTSD, others were likely in remission. As previously noted by Cornelis and colleagues [11], genetic influences may differ for current vs. lifetime PTSD. They suggested that making the distinction between lifetime and current PTSD in genetic studies may be important for case definition. In our review, only 18 articles reported whether participants had current or lifetime PTSD. Of the 18 available, seven included participants with only current PTSD. Further complicating the issue of caseness, the method and criteria by which PTSD was assessed varied markedly. Some studies used self-report questionnaires [47], whereas others used formal clinical interviews [56].

PTSD itself is a heterogeneous phenotype. Comprised of 17 symptoms, several of the symptoms—like Cluster B’s intense distress at reminders of trauma and Cluster C’s feelings of numbness—are markedly distinct from each other. Empirical investigation into the distinct symptom presentations has indicated that individuals diagnosed with PTSD often have heterogeneous clinical presentations [58,59]. In the context of a review on PTSD’s genetic underpinnings, the idiosyncrasies in symptom presentations raise questions about the underlying genetic mechanisms. As the symptom phenotypes can be markedly distinct, it is possible that their corresponding genetic substrates would also be different. Findings from some of the studies are consistent with this hypothesis. Dragan and colleagues found that at least one copy of the *DRD4* long allele related to Avoidance/Numbing scale (and Total PSTD score) but not to other symptom clusters. Likewise, Bailey and colleagues found moderate heritabilities of PTSD diagnosis and C category symptoms, and high heritabilities of B symptom categories [60].

The third major methodological limitation relates to sample size. Virtually all studies were obstructed by insufficient sample sizes. Factors impacting power to detect main genetic effects will also apply to tests for G × E interactions. The prevalence and effect of the environmental pathogen, as well as the type and size of interaction effect will also determine study power. A heuristic is that a fourfold increment in sample size is needed to examine multiplicative interactions between two main effects [61].

As a result of the aforementioned limitations (i.e., operationalization of trauma, PTSD caseness and sample size), different configurations of genetic risk based on either allele or genotype, and the direction of the association, there are contradictory results across various studies. For example, Segman and colleagues found that DAT is related to PTSD, while Bailey and colleagues did not. In the first study, the authors showed a significant association of the homozygote genotype for the 9 repeats allele (9/9) with PTSD, while in the second study a simple allelic association of the 9 repeats allele was tested and did not reach statistical significance. Likewise, Gelernter and colleagues [43] did not find any association between DRD2 and PTSD, while Comings and colleagues did [42]. In the first study, the authors reported a lack of association when comparing D2A1 carriers (i.e., D2A1/ D2A1 plus D2A1/ D2A2 subjects) and D2A2 homozygotes. In the conclusions of Comings and colleagues, the positive finding was reported for the allelic association with PTSD of D2A1 carriers as opposed to the non-carriers. Even more complex is the scenario of association studies on the 5-HTTLPR polymorphism of the serotonin transporter (SLC6A4). Different configurations of the genetic risk were considered in the analyses. Some studies compared the rates of the three genotypes ss/sl/ll [36,46,53]; others tested differences between the group of carriers of the ll genotype and the group of sl and ll genotype carriers [40]. Yet still others considered allelic associations--either s or l alleles—separately [62]. Several studies modeled the genotypic configuration based on the independent contribution of the 5-HTT functional expression alleles, which groups the s and lg (i.e. the diplotype constructed with the l allele of 5-HTTLPR and the g allele of the A/G SNP rs25531 within the 5-HTTLPR insertion) as the low expression functional alleles [63,64]. Differences in the direction of the allelic association have also been reported. Some studies reported association of the s allele with PTSD [29,57], while others the l allele [62]. A meta-analysis would be highly recommended to derive more robust conclusions about the association of 5-HTTLPR with PTSD. Overall, these examples underlie that determining whether the inconsistencies across studies are a result of differences in the genetic risk definition or a true lack of replication remains a challenge [65]. Ultimately, these contradictory results underscore the need to attend to these differences for not only interpretative purposes but also as a method of progress in the field of PTSD genetics. Despite its limitations, the available literature does raise compelling questions about the importance of studying the GxE relationship in the context of PTSD.

### Current challenges & future directions

It is hoped that the review of these 28 articles has illuminated important questions that future studies may seek to answer. In addition to the four aforementioned conclusions that were predicated on information included in these 28 studies, this review also offers two main guidelines for future studies. First, we suggest a framework for future studies that will allow for the systematic examination of data in a more standardized way. Specifically, an analytical approach that clearly provides information on the GxE interaction as well as the main effects of both trauma and genotype on PTSD would be of great benefit in gaining a more precise understanding of nature of this relationship. Likewise, future studies should also consider the effects of ethnic differences in allele frequency and, subsequently, the consequences that such population stratification may have on understanding the risk for and etiology of PTSD.

Second, it is also hoped that the findings from this review inform future study in this area. For example, future hypothesis-free, genome-wide genetic studies will be of great import in fostering a deeper understanding of PTSD’s etiology. Additionally, studies will be aided by incorporating more precise measures of environmental factors. For example, more explicit information on: 1) the specific nature of the index trauma; 2) when the index trauma occurred in the context of development; 3) the number of lifetime traumas, and 4) the chronicity versus acuity of PTSD in the sample would represent an important contribution in our understanding of environmental stress and, subsequently, how it interfaces with genotype. Moreover, advances in our understanding of the relationship between genetics and environment will be enhanced by future studies that explore—not only the GxE relationship—but also the more complex GxGxE and GxExE relationships. Exploration of interactions beyond the GxE relationship will almost certainly elucidate more about PTSD’s intricate and multifactorial etiology. Likewise, efforts to elucidate the causality in these interactions as well as the molecular mechanisms behind them will do much to further our understanding of the psychopathology. Finally, functional studies that examine the downstream consequences of the GxE interaction on biological pathways will also aid in our understanding of the etiology. Although the clinical applications of GxE research are presently remote, gaining a deeper understanding of how genetic and environmental risk factors contribute to the disorder will allow for more effective predictions, evaluations and, ultimately, treatment of PTSD.

## Competing interests

The authors declare that they have no competing interests.

## Authors’ contributions

All authors read and approved the final manuscript.
